# 

**Published:** 1890-08-30

**Authors:** 


					price one penny.
r? Vol. vm.-
-August 30th, 1890.
t **ed a.t the General Post Office as a N ewspaper for
^snuBsion in the United Kingdom and Abroad,]
WITH EXTRA NURSING SUPPLEMEP?T-
Per Annum, 6s. 6d.; post free.
Monthly Parts, 6<L; post free 8d.
Ditto per Annum. 8s.. post free.
SATURDAY, AUGUST 30th, 1890.

				

